# Icotinib is as efficacious as gefitinib for brain metastasis of EGFR mutated non-small-cell lung cancer

**DOI:** 10.1186/s12885-020-6543-y

**Published:** 2020-01-30

**Authors:** Kejun Liu, Guanming Jiang, Ailing Zhang, Zhuanghua Li, Jun Jia

**Affiliations:** 1 Department of Oncology, Dongguan Institute for Clinical Cancer Research, Dongguan People’s Hospital, Southern Medical University, 3 Wandao Road South, Dongguan, 523059 Guangdong China; 2 Department of Galactophore, Dongguan Institute for Clinical Cancer Research, Dongguan People’s Hospital, Southern Medical University, Dongguan, China

**Keywords:** Brain metastasis, EGFR mutation, Targeted therapy, Non-small-cell lung cancer (NSCLC)

## Abstract

**Background:**

The prognosis of non-small-cell lung cancer (NSCLC) with brain metastases is very poor. Currently, therapeutic methods for this patient population include whole-brain radiation therapy (WBRT), surgery, radiosurgery and systemic treatment. Epidermal growth factor receptor tyrosine kinase inhibitors (EGFR-TKIs) could be effective on cerebral metastases of mutated NSCLC. However, which EGFR-TKIs is more appropriate is still unknown.

**Methods:**

We conducted a retrospective analysis of advanced NSCLC patients with brain metastases for EGFR targeted therapy from November 2013 to April 2018 at Dongguan People’s Hospital, Southern Medical University, China. A total of 43 patients were recruit in this study. Among them, 21 cases received icotinib (125 mg, thrice a day) and 22 cases received gefitinib (250 mg, once a day) until disease progression or unacceptable toxicity. The primary end point of this study was intracranial PFS (iPFS). The relationships between therapeutic arms and patients characteristics were performed using Pearson’s chi-square test or Fisher’s exact test. The differences in PFS among the two arms were analyzed using Kaplan-Meier curves and log rank tests.

**Results:**

There was no significant difference of intracranial ORR (66.6% versus 59.1%, *P* = 0.62) and DCR (85.7% versus 81.8%, *P* = 0.73) between the two arms. The median intracranial PFS (iPFS) for icotinib and gefitinib arms were 8.4 months (95% CI, 5.4 to 11.3 months) and 10.6 months (95% CI, 6.3 to 14.8 months), respectively (*P* = 0.17). Adverse events of the two study arms were generally mild. None of the patients experienced dose reduction of EGFR-TKIs.

**Conclusions:**

Our study showed that icotinib and gefitinib had similar efficacy for brain metastasis of EGFR mutated NSCLC. Large randomized studies are suggested to further illuminate the effect of these two EGFR-TKIs on cerebral lesions of NSCLC.

## Background

Non-small-cell lung cancer (NSCLC) accounts for approximately 80% of all cases of lung cancer and is the leading cause of deaths resulting from carcinomas [1]. About 20–40% NSCLC patients will eventually develop brain metastasis [2–4]. The median overall survival (OS) of patients with brain metastases without any treatment was no more than 3 months [5]. The therapeutic methods for brain metastases of NSCLC were very limited and the treatment outcomes are relatively poor [6].

Whole-brain radiation therapy (WBRT) was once the standard treatment for NSCLC patients with multiple brain metastases, with a median OS about 3–7 months [7–9]. However, the neurocognitive toxicity associated with WBRT, which occurs several months to years after treatment, is always a concern among doctors and patients. Surgery or radiosurgery with or without WBRT could be a treatment choice for NSCLC patients with one to three brain metastases [10, 11]. Nevertheless, for patients with poor performance status, surgery or radiosurgery may not be feasible. As to chemotherapy for patients with brain metastases, there are different considerations, mainly because of the impenetrable feature of blood-brain barrier (BBB) [12]. Previous studies showed that the integrity of BBB was disrupted when brain metastases occurred, suggesting that chemotherapeutic agents used for advanced NSCLC could move into cerebrospinal fluid, thus may play an important role on brain metastases [13, 14]. The response rates of first-line chemotherapy for brain metastases of NSCLC ranged from 23 to 50% for cisplatin based regimens [15–18].

It is reported that 40–50% cases of lung adenocarcinoma harboring epidermal growth factor receptor (EGFR) mutation in Asian patients [19]. EGFR-tyrosine kinase inhibitors (EGFR-TKIs), such as gefitinib and erlotinib, were proved to be highly effective and less toxic to treat this patient population, as compared to traditional chemotherapeutic regimens [20–22]. The median OS of advanced NSCLC harboring EGFR mutation was prolonged to approximately 2 years when receiving regularly targeted therapy. Prescription of EGFR-TKIs in the first-line setting could reduce the incidence rate of brain metastasis among NSCLC patients [23]. For those patients who already diagnosed with brain metastases, EGFR-TKIs may also exert antitumor efficacy for intracranial disease. A large number of studies investigated the efficacy of targeted therapy and brain metastases of EGFR mutated NSCLC and showed similarly response rates and acceptable side effect [24–27].

As is known to us, gefitinib and erlotinib could cross the BBB and may be used to improve the therapeutic effects of WBRT [28]. Previous study showed that there was no significant differences in efficacy between the two drugs for EGFR mutated NSCLC with brain metastases [29]. However, gefitinib was more extensively used in the clinical for relatives lower toxicity. Icotinib is an orally administered EGFR-TKI, which has the same efficacy as gefitinib for second-line use in advanced NSCLC [30]. In a recent study, researcher compared the efficacy and toxicity of icotinib with WBRT plus chemotherapy for first-line therapy of NSCLC patients. Results showed that icotinib exerted better intracranial efficacy, indicating that icotinib might be a choice for brain metastases of NSCLC [31].

To date, it is still unclear whether icotinib or gefitinib is more suitable for EGFR mutated NSCLC patients with brain metastases. Therefore, we conducted this retrospective study comparing efficacy and toxicity of icotinib and gefitinib for brain metastasis of NSCLC harboring EGFR mutation.

## Methods

### Patient selection

We conducted a retrospective analysis of advanced NSCLC patients with brain metastases for EGFR targeted therapy from November 2013 to April 2018 at Dongguan People’s Hospital, Southern Medical University, China. The eligible patients were ≥ 18 years old, with cytological or histological confirmation of stage IIIB (with pleural effusion) and stage IV NSCLC (The International Association for the Study of Lung Cancer 7th edition of Tumor Node Metastasis Staging classification), harboring EGFR sensitive mutation of Exon 19 del or Exon 21 L858R, with at least one measurable site of brain metastasis by Response Evaluation Criteria in Solid Tumors version 1.0 (RECIST) and treated with either icotinib or gefitinib. Patients excluded from our analysis including those who were allergic to icotinib or gefitinib, reluctant to accept oral drug treatment for brain metastasis, receiving other EGFR targeted therapy, during the pregnant, suffered from primary organ failure or owned unavailable follow-up data. Patients whose clinicopathological characteristics could not be acquired were also excluded from this retrospective study.

**Fig. 1:**
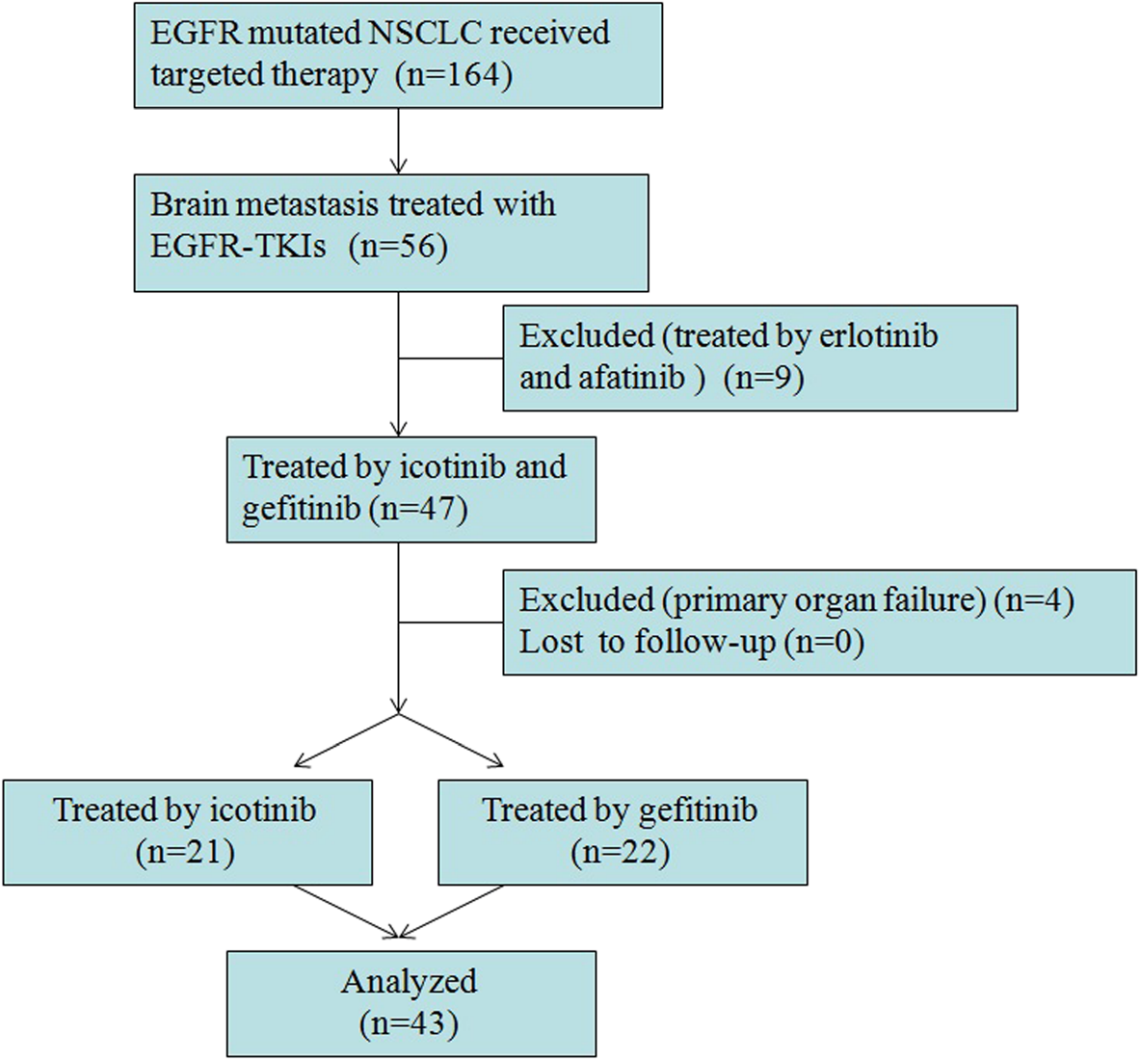
Patient selection flow diagram

There were 164 advanced NSCLC patients with EGFR mutation received targeted therapy. Among them, 56 patients were diagnosed with brain metastasis before the initiation of EGFR-TKIs treatment. A total of 47 patients were treated with icotinib and gefitinib, whereas 9 patients were treated by erlotinib and afatinib. For patients undergone icotinib and gefitinib therapy, 4 cases had primary organ failure, which is excluded at the beginning of this retrospective study. None of the 43 eligible patient were lost to follow-up and all of them were analyzed in our final evaluation for treatment efficacy and toxicity (Fig. 1).

### Treatment schedule

This retrospective study was approved by local ethics committees and was conducted according to the Declaration of Helsinki. Brain metastasis was confirmed by magnetic resonance imaging (MRI). EGFR mutation was identified in tumor tissues using the peptide nucleic acid-locked nucleic acid polymerase chain reaction clamp method (Sanger), the scorpion amplification refractory mutation system method (ARMS) or next-generation sequencing technology (NGS). A total of 43 NSCLC patients with brain metastasis harboring EGFR mutations were included in this study. Among these patients, 21 cases received icotinib (125 mg, thrice a day) and 22 cases received gefitinib (250 mg, once a day) until disease progression or unacceptable toxicity. Toxicities were recorded and classified according to the National Cancer Institute Common Terminology Criteria for Adverse Events (NCI-CTCAE) version 3.0. All patients provided informed written consent.

### Data collection

Clinical data of NSCLC patients with brain metastases were recorded carefully at baseline. EGFR-TKIs treatment time were also recorded in detail. Tumor response was evaluated in the light of Response Evaluation Criteria in Solid Tumors (RECIST) criteria. Disease control was defined as complete remission (CR), partial remission (PR) or stable disease (SD) or without progression disease (PD). PFS was defined as time between the start of EGFR targeted therapy and PD of NSCLC or death, with censoring for patients alive without progression at last contact. The cutoff date for PFS data was October, 2018, when the last patient had received targeted therapy for half of 1 year.

### Statistical analysis

The primary end point of this study was intracranial PFS (iPFS). The secondary end points were objective response rate (ORR) and disease control rate (DCR). All patients with EGFR-TKIs treatment were evaluable for response. Average response rate and 95% confidence interval were calculated separately for each arm of the study. The sample size was calculated by Power Analysis and Sample Size (PASS) 11.0 software. Statistical analysis was performed by Statistical Product and Service Solutions (SPSS) 22.0 software. The relationships between therapeutic arms and patients characteristics were performed using Pearson’s chi-square test or Fisher’s exact test. The differences in PFS among the two arms were analyzed using Kaplan-Meier curves and log rank tests. *P* values < 0.05 were considered as statistically significance.

## Results

### Baseline characteristics and treatment

The clinical characteristics of NSCLC patients in this study are listed in Table 1. The median age of icotinib arm was 63 years (range, 39–81 years), while that of gefitinib arm were 61 years (range, 41–79 years). Most patients had multiple brain metastases (90.5% versus 77.3%) and had never received chemotherapy (76.2% versus 90.9%). All patients had EGFR sensitive mutations, including Exon 19 del (47.6% versus 45.5%), Exon 21 L858R (52.4% versus 54.5%). There were 8 patients received brain radiation therapy during the initial treatment of targeted therapy, 5 in the icotinib arm and 3 in the gefitinib arm (23.8% versus 13.6%). Among these patients, only one patients received stereotactic radiotherapy. There were no statistically significant differences between the two arms of icotinib and gefitinib (Table 1). All patients received treatment of EGFR-TKIs, gefitinib (250 mg/day) or icotinib (375 mg/day). No major differences existed between the two arms concerning treatment time and dose reduction.

**Table 1:** Patients characteristics

Characteristic	Icotinib (*n* = 21)	Gefitinib (*n* = 22)	*P* value
Age			
Median	63	61	
Range	39 to 81	41 to 79	
Years			
< 65	12 (57.1%)	14 (63.6%)	0.66
≥ 65	9 (42.9%)	8 (36.4%)	
Sex			
Male	8 (38.1%)	2 (9.1%)	0.06
Female	13 (61.9%)	20 (90.9%)	
ECOG PS			
0–1	13 (61.9%)	16 (72.7%)	0.45
2 or more	8 (38.1%)	6 (27.3%)	
Smoking			
Yes	7 (33.3%)	2 (9.1%)	0.11
No	14 (66.7%)	20 (90.9%)	
Pleural effusion			
Yes	8 (38.1%)	9 (40.9%)	0.85
No	13 (61.9%)	13 (59.1%)	
Metastatic organs			
1	4 (19.1%)	3 (13.6%)	0.95
2 or more	17 (80.9%)	19 (86.4%)	
Number of brain metastases			
Single	2 (9.5%)	5 (22.7%)	0.45
Multiple	19 (90.5%)	17 (77.3%)	
Size of brain metastases			
≥ 20 mm	9 (42.9%)	4 (18.2%)	0.08
< 20 mm	12 (57.1%)	18 (81.8%)	
Brain radiation therapy			
Yes	5 (23.8%)	3 (13.6%)	0.64
No	16 (76.2%)	19 (86.4%)	
EGFR mutation status			
Exon 19 del	10 (47.6%)	10 (45.5%)	0.89
Exon 21 L858R	11 (52.4%)	12 (54.5%)	
Previous chemotherapy			
Yes	5 (23.8%)	2 (9.1%)	0.37
No	16 (76.2%)	20 (90.9%)	

*Abbreviations***:** *ECOG* Eastern Cooperative Oncology Group, *PS* physical score, *EGFR* epidermal growth factor receptor

### Efficacy

**Fig. 2:**
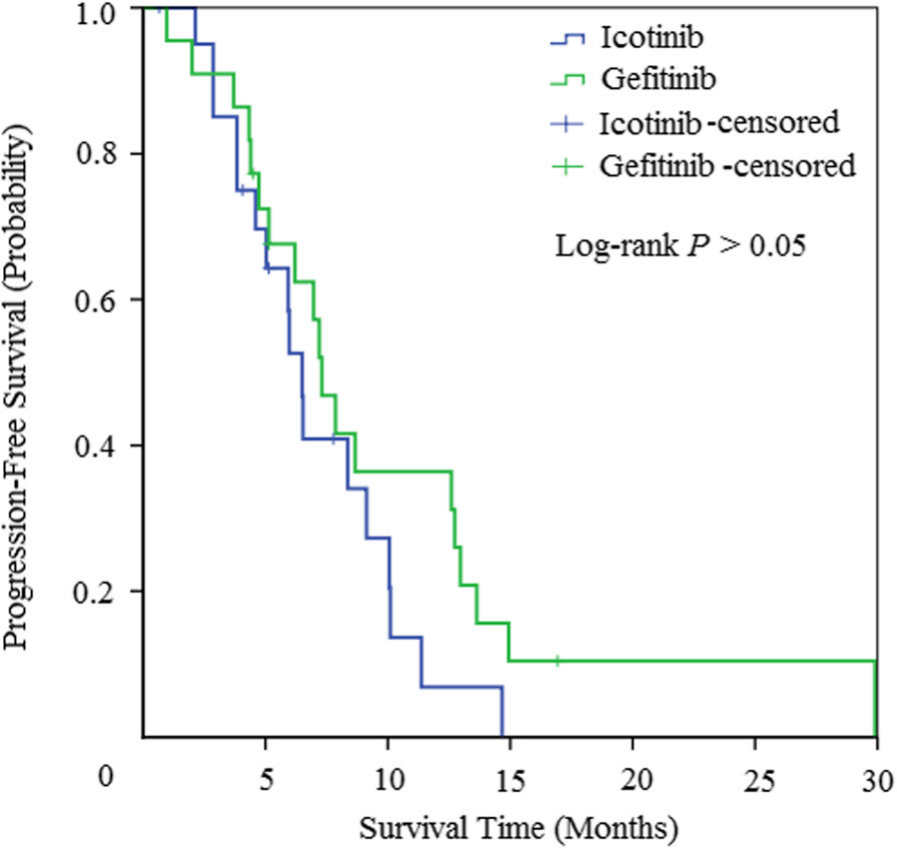
Kaplan-Meier curves for progression-free survival (PFS)

The response rate of NSCLC patients with brain metastases treated with icotinib was 57.1% (95% CI: 34.1 to 80.2), while that of gefitinib was 63.7% (95% CI: 41.8 to 85.5) (Additional file 1: Table S1). There was no significant difference in ORR or DCR between the two study arms (p**>**0.05), which is similar to former large randomized clinical trails. The median PFS of icotinib arm was 6.5 months (95% CI, 5.7 to 7.3 months), whereas that of gefitinib arm was 7.3 months (95% CI, 6.1 to 8.6 months) (Fig. 2). There was still no significant difference between the two study arms (*P* = 0.17).

**Fig. 3:**
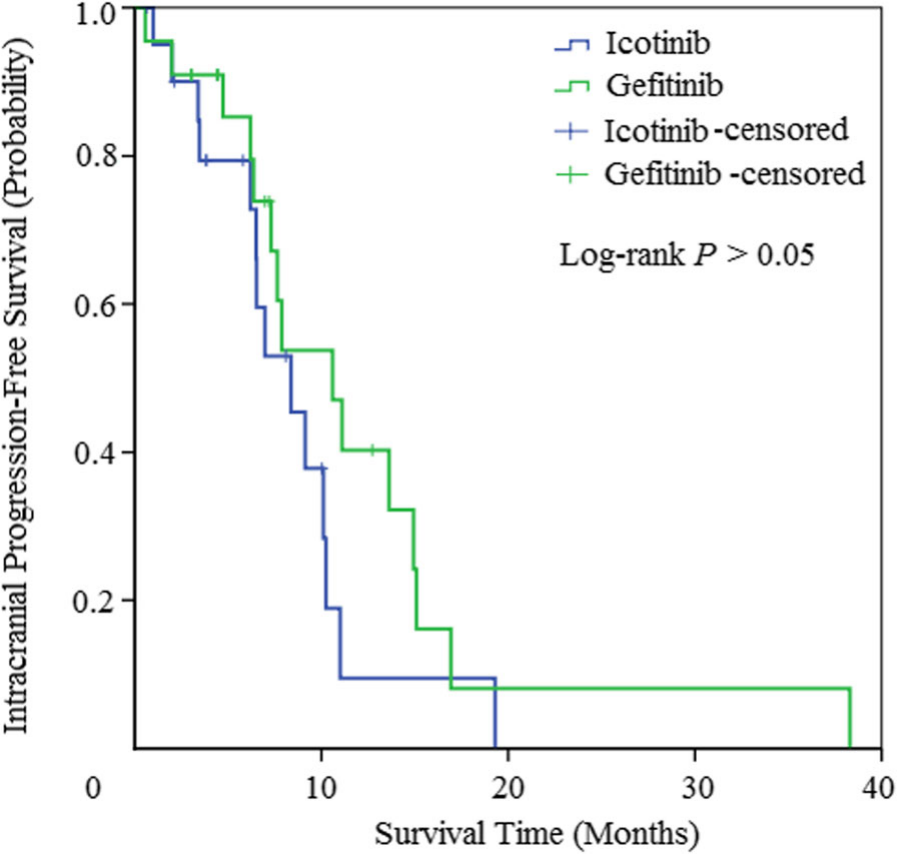
Kaplan-Meier curves for intracranial progression-free survival (iPFS)

The intracranial efficacy results of EGFR-TKIs treatment is illustrated in Table 2. In icotinib arm, there were 3 cases of CR, 11 cases of PR and 4 cases of SD. While that in gefitinib arm were 3 cases, 10 cases and 5 cases, respectively. There was no significant difference of intracranial ORR (66.6% versus 59.1%, *P* = 0.62) and DCR (85.7% versus 81.8%, *P* = 0.73) between the two arms. The median iPFS for NSCLC patient was relatively longer than the whole PFS of icotinib and gefitinib treatment, about 8.4 months (95% CI, 5.4 to 11.3 months) and 10.6 months (95% CI, 6.3 to 14.8 months), respectively, with no statistically significant difference among the two EGFR-TKIs treatment (*P* = 0.17) (Fig. 3).

**Table 2:** Intracranial efficacy sesults

Variable	Icotinib (*n* = 21)		Gefitinib (*n* = 22)		*P* value
	No.	%	No.	%	
Intracranial response					
CR (%)	3	14.3	3	13.6	
PR (%)	11	52.3	10	45.5	
SD (%)	4	19.1	5	22.7	
PD (%)	2	9.5	2	9.1	
NA (%)	1	4.8	2	9.1	
Intracranial RR, %	66.6.		59.1		0.62
95% CI	44.7 to 88.6		36.8 to 81.4		
Intracranial DCR, %	85.7		81.8		
95% CI	69.4 to 100		64.3 to 99.3		0.73
Median iPFS (months)	8.4		10.6		0.2
95% CI	5.4 to 11.3		6.3 to 14.8		

*Abbreviations*: *CR* complete remission, *PR* partial remission, *SD* stable disease, *PD* progression disease, *NA* not assessed, *RR* response rate, *DCR* disease control rate

### Adverse events

Main toxicities possibly related to icotinib and gefitinib treatment are listed in Table 3, including rash, pruritus, dizziness, fever, diarrhea, fatigue, nausea, vomiting, anorexia, raised aminopherase, dyspnea and hemorrhage, which were almost the same as what previous studies reported [21, 30]. Adverse events of the two study arms were generally mild. The most common grade 1/2 toxicities were rash (33.3% versus 40.9%), nausea (28.6% versus 31.8%) and pruritus (23.8% versus 27.3%). There was no statistical difference between arms of icotinib and gefitinib (p**>**0.05). A total of 4 cases of grade 3/4 adverse events occurred in this study, including 1 case of rash (4.8%) and 1 case of raised aminopherase (4.8%) in the icotinib arm and 2 cases of rash (9.1%) in the gefitinib arm. For adverse events of grade 3/4, there were still no significant statistical difference between the two arms (*p* > 0.05).

**Table 3:** Treatment related toxicities

Toxicity	Grade 1/2			Grade 3/4		
	Icotinib (*n* = 21)	Gefitinib (*n* = 22)	*P* value	Icotinib (*n* = 21)	Gefitinib (*n* = 22)	*P* value
Rash	7 (33.3%)	9 (40.9%)	0.62	1 (4.8%)	2 (9.1%)	0.59
Pruritus	5 (23.8%)	6 (27.3%)	0.80	0	0	
Dizziness	1 (4.8%)	2 (9.1%)	0.59	0	0	
Fever	1 (4.8%)	1 (4.5%)	0.97	0	0	
Diarrhea	1 (4.8%)	3 (13.6%)	0.33	0	0	
Fatigue	4 (19%)	3 (13.6%)	0.64	0	0	
Nausea	6 (28.6%)	7 (31.8%)	0.83	0	0	
Vomiting	2 (9.5%)	5 (22.7%)	0.25	0	0	
Anorexia	5 (23.8%)	6 (27.3%)	0.80	0	0	
Raised aminopherase	3 (14.3%)	5 (22.7%)	0.49	1 (4.8%)	0	0.31
Dyspnea	3 (14.3%)	4 (18.2%)	0.74	0	0	
Hemorrhage	1 (4.8%)	2 (9.1%)	0.59	0	0	

## Discussion

Around the world, there are 25–40% of patients suffered from brain metastases during the course of advanced NSCLC [32]. Metastasis to central nervous system, primarily in the cerebral hemisphere, is a severe complication of advanced NSCLC. The prognosis of such patients is generally poor, with a median survival ranging from 2 to 6 months in the past [33]. Treatment options for these patients before the era of targeted therapy were quite limited, including only WBRT, stereotactic radiosurgery, surgery and chemotherapy [34]. Although these therapeutic methods could be combined with each other, the efficacy results is not as good as enough. Importantly, traditional chemotherapeutical methods could lead to multiple side effects including nausea, emesis, anorexia and myelosuppression. What is more, neurocognitive dysfunction and declines in quality of life is unavoidable for certain patients receiving WBRT treatment, which occurred in several months to years after initial cerebral radiotherapy [35, 36]. Thus, novel treatment strategy with relatively tolerable toxicity is urgently needed.

As is known to us, targeted therapy could obtain better extra-cranial antitumor efficacy as compared to chemotherapy for EGFR mutated NSCLC, mainly due to inhibition of the corresponding signaling pathway. So far, there is enough evidence to show that the efficacy of gefitinib is parallel to that of erlotinib for treatment of brain metastases [37]. However, we still do not know whether other first generation of EGFR-TKIs could produce comparable therapeutic effect. Hence, we conducted this study comparing icotinib, one of the most frequently prescribed EGFR targeted agent in China, with gefitinib for cerebral metastases of NSCLC.

As the ICOGEN study showed to us, the efficacy of icotinib is non-inferiority when compared with gefitinib for patients with NSCLC beyond second-line treatment (median PFS 4.6 versus 3.4 months, HR 0.84) [30]. In this study, 24 patients (12%) diagnosed with brain metastases in the icotinib arm, while this patients number in the gefitinib arm is 26 (13%). Nevertheless, this study did not further analyze the effect of these two EGFR-TKIs on brain metastases of NSCLC. In the BRAIN study, researchers found that icotinib for first-line treatment could bring out significantly longer intracranial PFS than WBRT plus chemotherapy (median iPFS 10.0 versus 4.8 months, HR 0.56), suggesting that icotinib might be a better treatment method for NSCLC patients with brain metastases [31]. Still, we do not know which EGFR-TKI is more appropriate for target treatment of selected NSCLC patient harboring cerebral lesions.

In our study, we found that the whole efficacy and toxicity between icotinib and gefitinib were similar to each other (Additional file 1: Table S1). The results of response rates, disease control rates and median PFS in the two treatment arms were approximately 60, 85% and 7 months, respectively. These results were in accordance with former studies, which showed that the response rate of EGFR-TKIs for intracranial lesions was up to 88%, with a disease control rate ranging from 27 to 100% [38, 39]. No obvious difference concerning median PFS existed between the two EGFR-TKIs (Fig. 2). For brain metastases, the intracranial response rate of icotinib arms was 66.6%, with 14.3% case of complete response, whereas that in gefitinib arm is 59.1 and 13.6% (Table 2). The median iPFS was 8.4 and 10.6 months, respectively, which is also similar to the previous results of EGFR-TKIs for cerebral metastases. There was also no statistically significant difference (Fig. 3).

Currently, several studies have already reported the efficacy of icotinib and gefitinib for preventing brain metastasis from advanced NSCLC patients with EGFR mutation. In one study, icotinib was compared with chemotherapy as first-line therapy of advanced lung adenocarcinoma [40]. A total of 131 patients with EGFR mutation were treated in the icotinib group. Result showed that the cumulative risk of brain metastasis was lower in the icotinib group. In another study**,** the effect of gefitinib on EGFR mutated NSCLC with brain metastasis was evaluated [41]. There were 30 eligible patients received gefitinib according to the therapeutic strategy. It is revealed that gefitinib is effective for treating this subtype of NSCLC with cerebral metastasis. Based on the results of above studies, we conclude that EGFR-TKIs alone are effective for treatment of brain metastasis of EGFR mutated NSCLC. Nevertheless, we still do not know whether each of the first generation of EGFR-TKIs function similarly for these NSCLC patients. Our study confirmed that icotinib is as efficacious as gefitinib for brain metastasis of advanced NSCLC with EGFR mutation.

However, icotinib or gefitinib as single therapy may not be enough for EGFR-mutated NSCLC patients with cerebral metastasis, as compared with osimertinib, which has shown favorable intracranial efficacy for such patients [42]. Combined treatment is needed in order to improve the efficacy of first generation of EGFR targeted therapy. Several studies showed that EGFR-TKIs plus radiation therapy could produce better efficacy [43, 44]. The combination of icotinib or gefitinib with chemotherapy or anti-angiogenesis therapy may also enhance the treatment results of first generation of EGFR-TKIs, especially for those patients who can not afford the treatment of osimertinib.

As for treatment related toxicities, there is still no obvious difference between icotinib and gefitinib. There were only 4 cases of grade 3/4 adverse events occurred in this study, 2 in the icotinib arm and 2 in the gefitinib arm. These results indicated that icotinib was as efficacious as gefitinib for brain metastasis of EGFR mutated NSCLC, with generally mild toxicities. The toxicity profile in our study was also in accordance with the ICOGEN and BRAIN studies. No other side effects related with the two EGFR-TKIs were observed during the treatment procedure. Both icotinib and gefitinib were well tolerable among NSCLC patients with cerebral metastases. None of the 43 patients investigated in the study experienced dose reduction of EGFR-TKIs. Furthermore, since icotinib is cheaper than any other targeted agents such as gefitinib, erlotinib and afatinib, physicians would be more prone to prescribe icotinib for initial treatment of NSCLC in the clinic. In a word, it is suggested that icotinib might be an ideal therapeutic option for this patient population who is intolerable for severe toxicity as a result of cerebral lesions.

In this study, we also noted that the intracranial efficacy of icotinib was superior than the whole efficacy for NSCLC. This phenomenon was mainly due to the presence of BBB. As we all know, an intact BBB could reduce intracranial drug uptake of chemotherapeutic agents and certain EGFR-TKIs. Furthermore, multidrug resistance related protein such as ATP binding cassette (ABC) transporters is also located at the surface of BBB. It is confirmed that icotinib is one of the substrates of ABC transporters [45]. Therefore, the cerebrospinal fluid (CSF) concentrations may be lower than the corresponding plasma concentrations, which in turn caused delayed secondary resistance mutation in brain metastases as compared to the extra-cranial sites. Ultimately, the median iPFS was longer than the whole PFS with icotinib treatment. The similar results regarding median PFS were also observed in the gefitinib arm. On the contrary, the intracranial ORR and DCR is inferior as compared to the whole efficacy in our study. The specific reason is remaining uncertain. Basic research is needed to clarify the mechanism of this phenomenon.

## Conclusions

Our study showed that icotinib and gefitinib may have similar efficacy for brain metastasis of EGFR mutated NSCLC. Nonetheless, this is a retrospective analysis with a small number of patients. We did not make subtype analysis such as smoking status, EGFR mutation status and number of brain metastases. Large randomized studies are suggested to further illuminate the effect of these two EGFR-TKIs on cerebral lesions of NSCLC.

## Supplementary information

**Additional file 1: Table S1.** Efficacy Results.

## Data Availability

The datasets used and analyzed during the current study are available from the corresponding author on reasonable request.

## Abbreviations

ABC: ATP binding cassette
ARMS: The scorpion amplification refractory mutation system method
CR: Complete remission
ECOG: Eastern Cooperative Oncology Group
EGFR: Epidermal growth factor receptor
NCI-CTCAE: National Cancer Institute Common Terminology Criteria for Adverse Events
NGS: Next-generation sequencing technology
NSCLC: Non-small-cell lung cancer
ORR: Objective response rate
PD: Progression disease
PFS: Progression-free survival
PR: Partial remission
PS: Performance status
RECIST: Response evaluation criteria in solid tumors
SD: Stable disease
SPSS: Statistical product and service solutions
TKIs: Tyrosine kinase inhibitors
