# Supplementary material for: Icotinib is as efficacious as gefitinib for brain metastasis of EGFR mutated non-small-cell lung cancer
Source: BMC Cancer. 2020 Jan 30;20:76. doi: 10.1186/s12885-020-6543-y (PMC6993327; doi:10.1186/s12885-020-6543-y)
Supplement: Supplementary file 1 — Additional file 1: Table S1. Efficacy Results. [file 12885_2020_6543_MOESM1_ESM.doc]

Supplementary Table 1. Efficacy Results.

|  | Icotinib  (n=21) | Gefitinib  (n=22) | P value |
| --- | --- | --- | --- |
| Variable | No. % | No. % |  |
| Response |  |  |  |
| CR (%) | 0 | 1 4.5 |  |
| PR (%) | 12 57.1 | 13 59.2 |  |
| SD (%) | 5 23.8 | 5 22.7 |  |
| PD (%) | 3 14.3 | 1 4.5 |  |
| NA (%) | 1 4.8 | 2 9.1 |  |
| Response rates, % | 57.1. | 63.7 . | 0.67 |
| 95% CI | 34.1 to 80.2 | 41.8 to 85.5 |  |
| Disease control rates, % | 80.9 . | 86.4 | 0.64 |
| 95% CI | 62.6 to 99.3 | 70.8 to100 |  |
| Median PFS (months) | 6.5 . | 7.3 . | 0.17 |
| 95% CI | 5.7 to 7.3 | 6.1 to 8.6 |  |
